# Real-world clinical predictors of manic/hypomanic episodes among outpatients with bipolar disorder

**DOI:** 10.1371/journal.pone.0262129

**Published:** 2021-12-31

**Authors:** Keita Tokumitsu, Yasui-Furukori Norio, Naoto Adachi, Yukihisa Kubota, Yoichiro Watanabe, Kazuhira Miki, Takaharu Azekawa, Koji Edagawa, Eiichi Katsumoto, Seiji Hongo, Eiichiro Goto, Hitoshi Ueda, Masaki Kato, Atsuo Nakagawa, Toshiaki Kikuchi, Takashi Tsuboi, Koichiro Watanabe, Kazutaka Shimoda, Reiji Yoshimura

**Affiliations:** 1 Department of Psychiatry, Dokkyo Medical University School of Medicine, Tochigi, Japan; 2 The Japanese Society of Clinical Neuropsychopharmacology, Tokyo, Japan; 3 The Japanese Association of Neuro-Psychiatric Clinics, Tokyo, Japan; 4 Department of Neuropsychiatry, Kansai Medical University, Osaka, Japan; 5 Department of Neuropsychiatry, Keio University School of Medicine, Tokyo, Japan; 6 Department of Neuropsychiatry, Kyorin University School of Medicine, Tokyo, Japan; 7 Department of Psychiatry, University of Occupational and Environmental Health, Fukuoka, Japan; Federico II University of Naples, ITALY

## Abstract

**Background:**

Bipolar disorder is a mental illness in which manic and depressive states are repeated, causing psychosocial dysfunction. Manic/hypomanic episodes cause problems with interpersonal, social and financial activities, but there is limited evidence regarding the predictors of manic/hypomanic episodes in real-world clinical practice.

**Methods:**

The multicenter treatment survey on bipolar disorder (MUSUBI) in Japanese psychiatric clinics was administered in an observational study that was conducted to accumulate evidence regarding bipolar disorder in real-world clinical practice. Psychiatrists were asked to complete a questionnaire about patients with bipolar disorder who visited 176 member clinics of the Japanese Association of Neuro-Psychiatric Clinics by conducting a retrospective medical record survey. Our study extracted baseline patient characteristics from September to October 2016, including comorbidities, mental status, duration of treatment, Global Assessment of Functioning (GAF) score, and pharmacological treatment details. We investigated the presence or absence of manic/hypomanic episodes over the course of one year from baseline to September-October 2017.

**Results:**

In total, 2231 participants were included in our study, 29.1% of whom had manic/hypomanic episodes over the course of one year from baseline. Binomial logistic regression analysis revealed that the presence of manic/hypomanic episodes was correlated with lower baseline GAF scores, rapid cycling, personality disorder, bipolar I disorder, and a mood state with manic or mixed features. Substance abuse was also a risk factor for manic episodes. There was no significant association between a baseline antidepressant prescription and manic/hypomanic episodes.

**Conclusions:**

In Japan, 29.1% of outpatients with bipolar disorder had manic/hypomanic episodes over the course of one year. Our study suggested that a low GAF score, rapid cycling, personality disorder, bipolar I disorder, substance abuse, and baseline mood state could be predictors of manic/hypomanic episodes. Based on our findings, an antidepressant prescription is not a predictor of manic/hypomanic episodes.

## Introduction

Bipolar disorder is an affective disorder that results in chronic repeated severe mood changes with manic and depressive episodes; it is associated with characteristic cognitive, physical, and behavioral impairments [1–3]. Worldwide, the estimated lifetime prevalence of bipolar disorder among adults is 2.4% [4]. The prevalence is similar between men and women [5]. The average onset age for bipolar I disorder is 18 years, while that for bipolar II disorder is 20 years [4]. Inappropriate behavior caused by mood changes often has negative impacts on social, financial, and occupational outcomes [2]. Patients with bipolar disorder are 20 times more likely to commit suicide than the general population and bipolar disorder is associated with a high suicide rate [6].

Depression in patients with bipolar disorder is associated with long-term morbidity and impaired function [7]. The manic state is associated with increased medical costs and inappropriate behavior that leads to social problems such as wasting money and having aggressive attitudes [6]. Previous studies have shown that mood stabilizers reduce the risk of manic-depressive episodes and that antidepressant monotherapy may increase the risk of manic episodes. The incidence of treatment-emergent mania is reported to be 11.8–30.9% depending on the study settings and the incidence of treatment-emergent mania was low with the combined prescription of lithium [8] and second-generation antipsychotics [9]. However, there is contradictory evidence about the effectiveness of antidepressants for bipolar disorder, and no consensus has yet been reached. The predictors of manic episodes in the real world remain unclear.

More than 90% of mood disorder patients in Japan are outpatients, and approximately 50% of them are treated at clinics belonging to the Japanese Association of Neuro-Psychiatric Clinics (JAPC) [10]. Given this context, a collaborative study (the MUlticenter treatment SUrvey on BIpolar disorder in Japanese psychiatric clinics, or MUSUBI) was performed by the JAPC and the Japanese Society of Clinical Neuropsychopharmacology (JSCNP) to accumulate evidence on the real-world treatment of bipolar disorder in clinical practice in Japan [10–14]. In the MUSUBI study, we found that antidepressants are prescribed to a high proportion of patients with bipolar disorder (40.9%) [10], but the risk of manic/hypomanic episodes associated with the use of antidepressants could not be fully elucidated because of the cross-sectional design of the study. Antidepressant-induced mania/hypomania is a controversial issue that requires further research in not only clinical studies in specific settings but also real-world clinical practice. Therefore, we investigated the occurrence of manic/hypomanic episodes during the one year after baseline, focusing not only on baseline clinical features but also on each class of antidepressant prescribed to patients with bipolar disorder.

## Subjects and methods

### Study design and subjects

The MUSUBI study was a retrospective study in which a questionnaire was administered at 176 outpatient clinics belonging to the JAPC, with baseline patient characteristics collected from September to October 2016 [6]. We investigated the occurrence of manic/hypomanic episodes over the course of one year from baseline to September-October 2017. Patients diagnosed with bipolar disorder based on the ICD-10 criteria [3] and treated at these clinics were included in this study.

### Study procedures

Clinical psychiatrists were asked to complete a semi-structured questionnaire on patients with bipolar disorder by performing a retrospective medical record survey. The questionnaire included patient characteristics (age, sex, height, weight, academic background, and occupational status), comorbidities, mental status, Global Assessment of Functioning (GAF) score, and details of pharmacological treatment as the baseline data. In addition, we assessed the occurrence of manic/hypomanic episodes over the course of the year after baseline.

### Statistical analysis

All statistical analyses were performed with EZR (Saitama Medical Center, Jichi Medical University, Saitama, Japan) [15], which is a graphical user interface for R (The R Foundation for Statistical Computing, Vienna, Austria, version 3.5.2). More precisely, it is a modified version of R Commander (version 2.5–1) that incorporates statistical functions that are frequently used in biostatistics.

All statistical tests were two-sided, with a significance level of 0.05. Demographic and clinical characteristics were analyzed using the chi-square test and the Mann-Whitney U test to identify differences between patients with and without manic/hypomanic episodes over the course of the year after baseline. Univariate logistic regression analyses were performed to assess the demographic and clinical features. All factors associated with the occurrence of manic/hypomanic episodes among the bipolar disorder patients were identified using binomial logistic regression with forced entry to avoid overlooking any potential associations. These factors included sex, body mass index (BMI), age at study entry, age at the onset of bipolar symptoms, current employment status, education level, mood stabilizer prescription (valproic acid, lithium carbonate, carbamazepine and lamotrigine), antipsychotic prescription, anxiolytic (benzodiazepines only) prescription, hypnotic prescription, intelligence quotient (IQ), GAF score, psychiatric comorbidities, personality disorder, developmental disorder, physical comorbidities, rapid cycling status, substance abuse, suicidal ideation, types of bipolar disorder and mood status.

The following variables were included: male = 1, female = 2; employed = 1, unemployed = 0; Special support education school = 0, Junior high school = 1, High school, vocational school = 2, Junior college, technical college = 3, University = 4, Master’s degree or higher = 5; taking mood stabilizers = 1, not taking mood stabilizers = 0; taking antipsychotics = 1, not taking antipsychotics = 0; taking anxiolytics = 1, not taking anxiolytics = 0; taking hypnotics = 1, not taking hypnotics = 0; IQ (>85) = 0, IQ (85–71) = 1, IQ (<71) = 2; GAF (81–100) = 0, GAF (61–80) = 1, GAF (41–60) = 2, GAF (<41) = 3 (the actual GAF scores and the dummy variable of GAF scores in this study are inversely correlated); psychiatric comorbidity = 1, no psychiatric comorbidity = 0; personality disorder = 1, no personality disorder = 0; developmental disorder = 1, no developmental disorder = 0; physical comorbidity = 1, no physical comorbidity = 0; rapid cycling = 1, no rapid cycling = 0; substance abuse = 1, no substance abuse = 0; bipolar I disorder = 1, bipolar II disorder = 2; and suicidal ideation = 1, no suicidal ideation = 0. Four mood states (depressive, mixed, remission and manic) were analyzed as nominal measures. A bipolar disorder not otherwise specified was listwise deleted; patients with serious physical conditions such as terminal cancer or intractable diseases were excluded. Cases with missing values in the questionnaire responses were excluded from the study. Other factors do not exclude patients in this study. In addition, we also examined the relationship between baseline antidepressant prescription and the occurrence of manic/hypomanic episodes during the one year after baseline.

### Ethics

This study was conducted in accordance with the Declaration of Helsinki and the Japanese Ethical Guidelines for Medical and Health Research Involving Human Subjects. Prior to the initiation of the study, the study protocol was reviewed and approved by the institutional review board of the ethics committee of JAPC and Dokkyo Medical University School of Medicine. Since this was a retrospective medical record survey, it was exempted from the requirement for informed consent; however, we released information on this research so that patients were free to opt out. Administrative permissions and licenses were acquired by our team to access the data used in our research. The ethics committee of the Japanese Association of Neuro-Psychiatric Clinics set restrictions on data sharing because the data contain potentially identifying or sensitive patient information. Please contact the institutional review board of the ethics committee when requesting data. Contact information for our ethics committee: The institutional review board of the ethics committee of the Japanese Association of Neuro-Psychiatric Clinics; Shibuya-ku, Yoyogi 1-38-2, Tokyo Metropolis, Japan, Postal Code 151–0053, Phone +81-3-3320-1423.

## Results

As baseline data, completed questionnaires on 3213 outpatients with bipolar disorder were returned from the 176 originally solicited outpatient facilities. A total of 982 outpatients were excluded with listwise deletions. Thus, data on a total of 2231 patients were included in this study. The characteristics of the subjects are shown in Table 1.

**Table 1:** Demographic and clinical data for participants.

			manic/hypomanic episode for the next one year				
Factors	Dummy variable	Total (N = 2231)	manic/hypomanic episodes(-) (N = 1537)	manic/hypomanic episodes(+) (N = 694)	p-value	Scale	Statistical method
Gender					0.02	nominal scale	chi-square test
Male: n(%)	1	1025 (45.9)	732 (47.6)	293 (42.2)			
Female: n(%)	2	1206 (54.1)	805 (52.4)	401 (57.8)			
BMI (median [range])		22.90 [14.50, 45.40]	22.90 [14.50, 45.40]	22.90 [14.60, 39.60]	0.068	interval scale	Mann-Whitney U-test
Age at study entry: (median [range])		50.00 [13.00, 94.00]	50.00 [15.00, 90.00]	51.00 [13.00, 94.00]	0.584	interval scale	Mann-Whitney U-test
Age at onset of bipolar symptoms: (median [range])		34.00 [10.00, 81.00]	34.00 [12.00, 81.00]	33.00 [10.00, 78.00]	0.279	interval scale	Mann-Whitney U-test
GAF score: n(%)					<0.001	ordinal scale	Mann-Whitney U-test
81–100	0	757 (33.9)	620 (40.3)	137 (19.7)			
61–80	1	1019 (45.7)	686 (44.6)	333 (48.0)			
41–60	2	399 (17.9)	206 (13.4)	193 (27.8)			
1–40	3	56 (2.5)	25 (1.6)	31 (4.5)			
Current work status: n(%)	0	744 (33.3)	464 (30.2)	280 (40.3)	<0.001	nominal scale	chi-square test
	1	1487 (66.7)	1073 (69.8)	414 (59.7)			
Intelligence Quotient, IQ: n (%)					0.391	ordinal scale	Mann-Whitney U-test
> 85	0	2140 (95.9)	1479 (96.2)	661 (95.2)			
85–71	1	73 (3.3)	48 (3.1)	25 (3.6)			
< 71	2	18 (0.8)	10 (0.7)	8 (1.2)			
Educational level: n(%)					0.086	ordinal scale	Mann-Whitney U-test
Special support education school	0	7 (0.3)	5 (0.3)	2 (0.3)			
Junior high school	1	102 (4.6)	67 (4.4)	35 (5.0)			
High school, vocational school	2	1037 (46.5)	709 (46.1)	328 (47.3)			
Junior college, technical college	3	202 (9.1)	131 (8.5)	71 (10.2)			
University	4	806 (36.1)	580 (37.7)	226 (32.6)			
Master’s degree or higher	5	77 (3.5)	45 (2.9)	32 (4.6)			
Rapid cycler: n (%)	0	1973 (88.4)	1450 (94.3)	523 (75.4)	<0.001	nominal scale	chi-square test
	1	258 (11.6)	87 (5.7)	171 (24.6)			
Suicidal ideation: n (%)	0	1990 (89.2)	1404 (91.3)	586 (84.4)	<0.001		
	1	241 (10.8)	133 (8.7)	108 (15.6)			
Mood stabilizer prescription: n (%)	0	349 (15.6)	253 (16.5)	96 (13.8)	0.129	nominal scale	chi-square test
	1	1882 (84.4)	1284 (83.5)	598 (86.2)			
Antidepressants prescription: n (%)	0	1300 (58.3)	872 (56.7)	428 (61.7)	0.032	nominal scale	chi-square test
	1	931 (41.7)	665 (43.3)	266 (38.3)			
Antipsychotics prescription: n (%)	0	1070 (48.0)	776 (50.5)	294 (42.4)	<0.001	nominal scale	chi-square test
	1	1161 (52.0)	761 (49.5)	400 (57.6)			
Anxiolytics prescription: n (%)	0	1418 (63.6)	974 (63.4)	444 (64.0)	0.82	nominal scale	chi-square test
	1	813 (36.4)	563 (36.6)	250 (36.0)			
Hypnotics prescription: n (%)	0	897 (40.2)	655 (42.6)	242 (34.9)	0.001	nominal scale	chi-square test
	1	1334 (59.8)	882 (57.4)	452 (65.1)			
Physical comorbidity: n (%)	0	1555 (69.7)	1091 (71.0)	464 (66.9)	0.056	nominal scale	chi-square test
	1	676 (30.3)	446 (29.0)	230 (33.1)			
Personality disorder: n (%)	0	2119 (95.0)	1478 (96.2)	641 (92.4)	<0.001	nominal scale	chi-square test
	1	112 (5.0)	59 (3.8)	53 (7.6)			
Psychotic symptoms: n (%)	0	2080 (93.2)	1455 (94.7)	625 (90.1)	<0.001	nominal scale	chi-square test
	1	151 (6.8)	82 (5.3)	69 (9.9)			
Psychiatric comorbidity: n (%)	0	1855 (83.1)	1314 (85.5)	541 (78.0)	<0.001	nominal scale	chi-square test
	1	376 (16.9)	223 (14.5)	153 (22.0)			
Types of bipolar disorders: n (%)					<0.001	nominal scale	chi-square test
Bipolar I disorder	1	794 (35.6)	464 (30.2)	330 (47.6)			
Bipolar II disorder	2	1437 (64.4)	1073 (69.8)	364 (52.4)			
Developmental disorder: n (%)	0	2110 (94.6)	1472 (95.8)	638 (91.9)	<0.001	nominal scale	chi-square test
	1	121 (5.4)	65 (4.2)	56 (8.1)			
Substance abuse: n (%)	0	2117 (94.9)	1482 (96.4)	635 (91.5)	<0.001	nominal scale	chi-square test
	1	114 (5.1)	55 (3.6)	59 (8.5)			
Mood status: n(%)					<0.001	nominal scale	chi-square test
Depressive state		885 (39.7)	617 (40.1)	268 (38.6)			
Manic state		189 (8.5)	68 (4.4)	121 (17.4)			
Remission		942 (42.2)	759 (49.4)	183 (26.4)			
Mixed state		215 (9.6)	93 (6.1)	122 (17.6)			

Abbreviations, BMI: Body Mass Index, GAF: Global Assessment Functioning.

Because 23 comparisons were made, a Bonferroni correction was applied, yielding a corrected significance criterion of p <0.0022.

The proportion of patients who experienced manic/hypomanic episodes over the course of the year after baseline was 29.1% (694/2231). The results of the univariate analysis are shown in Table 1. Because 23 comparisons were made, a Bonferroni correction was applied, yielding a corrected significance criterion of p <0.0022. Based on this threshold, the occurrence of manic/hypomanic episodes significantly differed among the groups stratified by GAF scores, rapid cycler status, suicidal ideation, antipsychotic prescription, employment status, personality disorder, psychotic symptoms, psychiatric comorbidity, types of bipolar disorders, developmental disorder, mood status, substance abuse and hypnotic prescription.

A binomial logistic regression analysis revealed that the group of patients with manic/hypomanic episodes during the year after baseline had significantly lower GAF scores (odds ratio; OR [95% CI] = 1.54 [1.30–1.83], p<0.001), a higher proportion of patients with rapid cycling (OR = 3.86 [2.84–5.24, p<0.001]), a higher proportion of patients with personality disorders (OR = 1.80 [1.02–3.17], p = 0.042]), a higher proportion of patients with bipolar I disorder rather than bipolar II disorder (OR = 0.55 [0.44–0.68], p<0.001) and a higher rate of patients with substance abuse disorders than those who did not suffer manic episodes. The groups in a manic state (OR = 4.54 [3.13–6.58], p<0.001) and a mixed state (OR = 2.32 [1.02–2.44], p = 0.039) had significantly higher proportions of patients with manic/hypomanic episodes than the group in a depressive state (Table 2). In a binomial logistic regression analysis with forced entry, multicollinearity between variables was not observed.

**Table 2:** Binomial logistic regression analysis of baseline factors for manic/hypomanic episodes during the year after baseline.

Factor	Odds ratio (95% CI)	p-value
Gender	1.21 (0.97–1.51)	0.096
BMI	1.01 (0.98–1.04)	0.570
Age at study entry	1.00 (0.99–1.01)	0.600
Age at onset of bipolar symptoms	1.00 (0.99–1.02)	0.480
Current work status	0.91 (0.72–1.14)	0.400
Educational level	1.08 (0.98–1.20)	0.120
Mood stabilizer prescription	1.07 (0.81–1.43)	0.630
Antipsychotics prescription	1.08 (0.87–1.33)	0.490
Anxiolytics prescription	0.94 (0.76–1.17)	0.590
Hypnotics prescription	1.03 (0.84–1.28)	0.760
Antidepressants prescription	0.87 (0.70–1.08)	0.190
IQ	0.97 (0.65–1.47)	0.900
GAF score	1.54 (1.30–1.83)	<0.001
Psychiatric comorbidity	0.85 (0.56–1.30)	0.450
Personality disorder	1.80 (1.02–3.17)	0.042
Developmental disorder	1.52 (0.87–2.66)	0.140
Physical comorbidity	1.04 (0.83–1.30)	0.750
Rapid cycler	3.86 (2.84–5.24)	<0.001
Substance abuse	1.58 (1.02–2.44)	0.039
Suicidal ideation	1.13 (0.81–1.57)	0.480
Psychotic symptoms	1.02 (0.68–1.52)	0.930
Types of bipolar disorders	0.55 (0.44–0.68)	<0.001
Mood status (vs Depressive state)		
Manic state	4.54 (3.13–6.58)	<0.001
Remission	0.88 (0.66–1.15)	0.340
Mixed state	2.32 (1.65–3.26)	<0.001

Abbreviations, BMI: Body Mass Index, IQ: Intelligence Quotient, GAF: Global Assessment Functioning, CI: confidence interval.

We also analyzed the associations between baseline antidepressant prescriptions and the occurrence of manic/hypomanic episodes. Antidepressants were classified into selective serotonin reuptake inhibitors (SSRIs), serotonin–norepinephrine reuptake inhibitors (SNRIs), noradrenergic and specific serotonergic antidepressants (NaSSAs), serotonin antagonists and reuptake inhibitors (SARIs), and tricyclic antidepressants (TeCAs), and baseline mood status was stratified and analyzed. There was no significant association between a baseline antidepressant prescription and the occurrence of manic/hypomanic episodes during the one year after baseline (Table 3).

**Table 3:** The associations between baseline antidepressant prescriptions and the occurrence of manic/hypomanic episodes.

Factor	manic/hypomanic episodes (-)	manic/hypomanic episodes (+)	p-value
	n = 1537	n = 694	
NaSSA (%)	114 (7.4)	40 (5.8)	0.182
SARI (%)	81 (5.3)	37 (5.3)	1.000
SNRI (%)	180 (11.7)	62 (8.9)	0.060
SSRI (%)	277 (18.0)	125 (18.0)	1.000
TCA (%)	123 (8.0)	51 (7.3)	0.654
TeCA (%)	45 (2.9)	17 (2.4)	0.619

Abbreviations, NaSSA: Noradrenergic and Specific Serotonergic Antidepressant, SARI:

serotonin antagonist and reuptake inhibitors, SNRI: Serotonin Noradrenaline Reuptake

Inhibitor, SSRI: Selective Serotonin Reuptake Inhibitor, TCA: Tricyclic Antidepressants, TeCA: Tetracyclic Antidepressants.

## Discussion

Our study is the first multicenter study in Japan to investigate the predictors of manic/hypomanic episodes in outpatients with bipolar disorder. Importantly, in the binomial logistic regression analysis, we found that low baseline GAF scores, rapid cycling status, personality disorders, bipolar I disorder, substance abuse, and baseline mood states were predictors of manic/hypomanic episodes over the course of the year after baseline. This result cannot be generalized because the analysis was limited to Japanese outpatients, but manic/hypomanic episodes in patients with bipolar disorder can be predicted by considering multiple factors. Similarly, a previous study named the Systematic Treatment Enhancement Program for Bipolar Disorder (STEP-BD) reported that baseline manic state, rapid cycling status, substance abuse and suicide attempts were predictors of manic episodes [16]. Therefore, it is thought that manic episodes in patients with bipolar disorder can be predicted by combining certain factors regardless of culture or ethnicity.

In our study, the effect sizes of baseline manic state, mixed feature state and rapid cycling status were medium, indicating that they are relatively important predictors. Both the manic state and mixed feature state are characterized by manic features. It seems that a manic episode increases the risk of a subsequent manic episode. Interestingly, a review of placebo-controlled trials for bipolar disorder suggested that the baseline mood state of patients with bipolar disorder who entered the clinical trial was a predictor of their subsequent prognosis [17]. When the baseline state is a manic state, manic/hypomanic episodes are more likely to occur during the observation period [17]. Additionally, rapid cycling is specific to patients with bipolar disorder who have manic episodes more than four times per year. Past studies have shown that if rapid cycling is observed at baseline, there is an increased risk of subsequent manic episodes. [14, 18]. Rapid cycling is also associated with increased alcohol abuse and suicide, indicating that careful monitoring is needed [19].

Our study showed that patients with bipolar disorder with personality disorder were at a significantly elevated risk of experiencing mania/hypomanic episodes over the next year. The relationship between personality disorder and bipolar disorder has been discussed, especially in terms of the potential for comorbid borderline personality disorder and bipolar disorder [20]. It has been reported that approximately 1 in 5 patients with bipolar disorder have been diagnosed with comorbidities of borderline personality disorder [21]. Patients with bipolar disorder in real-world clinical settings commonly present with personality disorders [22]. A meta-analysis of 13 studies revealed that among 1101 outpatients and inpatients with bipolar disorder, at least one personality disorder was present in 42% [23]. In addition, compared to bipolar patients without a personality disorder, patients with comorbid personality disorders have a more severe course of illness, including a decreased likelihood of recovering from mood episodes [22, 24–25], worse psychosocial functioning and more suicide attempts [22]. Patients with borderline personality disorder often have difficulty controlling their emotions and impulses, which can lead to violent behavior or mood swings that are difficult to distinguish from manic episodes. In addition, due to unstable interpersonal relationships, anxiety, suicide attempts, and self-harm may occur, which may be perceived as a depressive episode. In this way, the phenomenological distinction between bipolar disorder and personality disorders may be difficult because of the overlap between the features of personality disorder and the diagnostic criteria for mood episodes [26]. A previous study reported that nearly 40% of borderline personality disorder patients were found to have a misdiagnosis of bipolar disorder [27], whereas other studies reported an even higher rate of the overdiagnosis of bipolar disorder [28]; therefore, a careful assessment is needed. Our study suggests that personality disorder may make it difficult for patients with bipolar disorder to maintain remission. Because manic episodes of bipolar disorder and the symptoms of personality disorder may affect each other, more careful follow-up is needed to achieve treatment success.

Previous reports have been published on the association between substance use disorders and bipolar disorder [26, 29]. The risk of bipolar disorder onset has been reported to increase with substance abuse such as alcohol abuse [6, 30]. In particular, the consumption of alcohol by patients with bipolar disorder increases their risk of manic episodes [30]. Some studies have shown that substance abuse is more likely to be associated with bipolar disorder, while others warn that patients with substance abuse are more likely to be overdiagnosed with bipolar disorder [31]. Substance abuse is also known to cause manic or depressive symptoms resembling those of bipolar disorder [26]. However, there is insufficient real-world evidence from patients with bipolar disorder with substance abuse disorders. We have shown that patients with bipolar disorder with substance abuse disorders are at a significantly elevated risk of experiencing a manic episode within a year. This is essential evidence that suggests that the treatment of comorbidities is important for patients with bipolar disorder.

We also found that when the baseline GAF score was low, manic/hypomanic episodes were more likely to occur in the next year. The DSM-5 stipulates that "the mood disturbance must be sufficiently severe to cause marked impairment in social or occupational functioning" for the diagnosis of manic episodes [32]. It is important to note that our study revealed that poor baseline social adaptation was a predictor of manic episodes. A previous study revealed that patients who had suffered more manic episodes had more cognitive dysfunction in the areas of verbal learning and memory [33]. Importantly, repeated and prolonged manic states impair cognitive function [34], and cognitive impairment itself is a predictor of poor prognosis in patients with bipolar disorder [35]. Treatment for cognitive dysfunction may improve the quality of life for patients with bipolar disorder by strengthening their academic performance, work capacity, and social relationships [6].

Our study indicated that patients with bipolar II disorder had a lower risk of developing manic/hypomanic episodes within a year than those with bipolar I disorder (OR = 0.55). In a previous 13-year long-term observational study, patients with bipolar II disorder were symptomatically ill in 53.9% of the weeks of follow-up. Patients had symptoms of depression in 50.3% of the weeks of follow-up and hypomania in 1.3% of the weeks of follow-up [36]. Patients with bipolar I disorder were reported to be symptomatically ill in 47.3% of the weeks of follow-up, with depressive symptoms in 31.9% of the weeks of follow‑up and manic or hypomanic symptoms in 8.9% of the weeks of follow‑up [37]. These studies found that bipolar II is characterized by a longer symptom duration (50.3% vs 47.3%), but manic episodes occur more frequently in bipolar I (8.9% vs 1.3%). The content of these previous studies is consistent with our findings that patients with bipolar I at baseline were more likely to have a manic/hypomanic episode over the course of the next year.

Interestingly, no significant relationship was found between an antidepressant prescription at baseline and the occurrence of manic/hypomanic episodes over the course of the next year. The results were similar when stratified by the baseline mood state. In addition, although there was no significant difference, the frequency of manic/hypomanic episodes tended to be lower in the group of patients prescribed SNRIs. Our study also demonstrated that SNRIs were the most commonly prescribed antidepressants among patients with bipolar disorder. The results of this study suggest the tolerability of antidepressants, particularly SNRIs, in patients with bipolar disorder. Recently, a systematic review and network meta-analysis of the efficacy and tolerability of pharmacological treatments for the treatment of acute bipolar depression was published; however, it did not include studies involving SNRIs [38]. The prescription of antidepressants for bipolar disorder remains a controversial issue. In particular, the risk of conversion to a manic state because of the use of antidepressants has been repeatedly reported [39, 40]. However, a recent real-world study reported that antidepressants are used by a large proportion of patients with bipolar disorder [39, 41–43]. While the treatment guidelines emphasize the high risks associated with tricyclic antidepressant monotherapy, they describe the effectiveness of antidepressant combination therapy for the treatment of acute depression and maintenance of remission [44]. In a study that analyzed the STEP-BD sample, antidepressant treatment was not found to be a primary risk factor for conversion from depression to mania in patients with bipolar disorder [16]. The presence of a manic episode before the onset of depression in patients with bipolar disorder did not significantly predict the response to antidepressants [45]. Recently, single-agent antidepressant prescriptions for bipolar II patients have also been accepted [46]. Bipolar disorder involves a long duration of depression, so the use of antidepressants may be acceptable if the risks and benefits of each individual case are carefully considered [46, 47]; however, there is insufficient high-quality evidence regarding the use of antidepressants by patients with bipolar disorder, and further supporting investigations are needed in the future.

### Limitations

Our study only covers outpatients, and information about inpatients remains unknown. Therefore, it does not reflect the entire population with bipolar disorder. In addition, since the study was conducted only in clinics that belonged to the Japanese Association of Neuro-Psychiatric Clinics and consented to participate in the study, there is a possibility that the composition of participants was biased. In addition, patient selection was not randomized and this was a retrospective study, which could have also led to selection bias. The results may differ in the long term, as follow-up in this study was limited to one year. Regarding the predictors of manic episodes, the presence or absence of antidepressants was analyzed based on the existence of a prescription at baseline. The durations of the prescriptions for various agents were not investigated. This is a limitation of our research, and further study is needed. Furthermore, we did not consider the severity of manic episodes and did not distinguish between manic and hypomanic episodes. We did not determine the exact number of people who experienced manic episodes (not hypomanic episodes). Therefore, the relationships between the severity of mania and the independent variables are unclear.

In addition, a previous review suggested problems with polypharmacy for patients with bipolar disorder [48]. Previously, our research group reported polypharmacy in a series of collaborative studies, and we found that the severity of illness and an intractable disease course were significantly associated with the number of psychotropic drugs in real-world clinical settings [13]. We performed multivariate analysis with the presence or absence of various psychotropic drugs (mood stabilizers, antipsychotics, antidepressants, hypnotics) as independent variables in the present study. In addition to these factors, adding the presence or absence of polypharmacy of the above drugs as an independent variable causes potential statistically multicollinear problems because each factor becomes a nested structure. For this reason, the risk of manic/hypomanic episodes due to polypharmacy was not fully understood.

Personality disorders include a variety of clinical conditions, but this study did not subdivide them, such as classifying clusters of personality disorders. Moreover, the odds ratio may not be adequately approximated to the risk ratio because the event rate of manic/hypomanic episodes was greater than 10% [49].

## Conclusions

In Japan, 29.1% of outpatients with bipolar disorder had manic/hypomanic episodes over the course of one year. Our study suggests that a low GAF score, rapid cycling, personality disorder, bipolar I disorder, substance abuse, and baseline mood state could be predictors of manic/hypomanic episodes. Based on our findings, antidepressant prescription is not a predictor of manic/hypomanic episodes.
