# Correction: Real-world clinical predictors of manic/hypomanic episodes among outpatients with bipolar disorder

**DOI:** 10.1371/journal.pone.0272758

**Published:** 2022-08-03

**Authors:** The PLOS ONE Staff

The fourth author’s name is spelled incorrectly in the author list. The publisher apologizes for the error. The correct name is: Norio Yasui-Furukori. The correct citation is: Tokumitsu K, Yasui-Furukori N, Adachi N, Kubota Y, Watanabe Y, et al. (2021) Real-world clinical predictors of manic/hypomanic episodes among outpatients with bipolar disorder. PLOS ONE 16(12): e0262129. https://doi.org/10.1371/journal.pone.0262129.
