# Supplementary material for: Real-world clinical predictors of manic/hypomanic episodes among outpatients with bipolar disorder
Source: PLoS One. 2021 Dec 31;16(12):e0262129. doi: 10.1371/journal.pone.0262129 (PMC8719757; doi:10.1371/journal.pone.0262129)
Supplement: Attachment — Submitted filename: Response to Reviewers.docx [file pone.0262129.s001.docx]

Authors’ response to the reviewers:

We are grateful to the reviewers for their critical comments and useful suggestions, which have helped us improve our paper. As indicated in the responses that follow, we have taken all these comments and suggestions into account in the revised version of our paper.

We hope that the revised version of our paper is now suitable for publication in *PLOS ONE*.

Sincerely,

Norio Yasui-Furukori, MD, PhD

PONE-D-21–18468

“Real-world clinical predictors of manic/hypomanic episodes among outpatients with bipolar disorder” (title was revised based on reviewers’ comments)

Journal Requirements:

Comment:

1. Please ensure that your manuscript meets PLOS ONE's style requirements, including those for file naming. The PLOS ONE style templates can be found at

https://journals.plos.org/plosone/s/file?id=wjVg/PLOSOne_formatting_sample_main_body.pdf and

<https://journals.plos.org/plosone/s/file?id=ba62/PLOSOne_formatting_sample_title_authors_affiliations.pdf>

Response:

Thank you for your guidance.

The manuscript has been revised according to *PLOS ONE's* style requirements.

Comment:

2. We noticed you have some minor occurrence of overlapping text with the following previous publication(s), which needs to be addressed:

- https://www.nature.com/articles/nrdp20188

- https://bmcpsychiatry.biomedcentral.com/articles/10.1186/s12888-020-02967-5

In your revision ensure you cite all your sources (including your own works), and quote or rephrase any duplicated text outside the methods section. Further consideration is dependent on these concerns being addressed.

Response:

Thank you for your important observation. We revised the duplicates in the manuscript and corrected the source citations.

(Lines 45, 81–84, 86–88, 96–98, 115-116, 122, 138, 304–305, 325–327)

Comment:

3. We note that the grant information you provided in the ‘Funding Information’ and ‘Financial Disclosure’ sections do not match.

When you resubmit, please ensure that you provide the correct grant numbers for the awards you received for your study in the ‘Funding Information’ section.

Response:

Our grant information is listed in the 'Funding Information' section instead of 'Financial Disclosure'. This study was supported by a Ken Tanaka memorial research grant (grant numbers: 2016–2, 2017–4 and 2019–3). The funder had no role in the study design, the data collection and analysis, the decision to publish, or the preparation of the manuscript. We added this statement to the ‘Funding Information’ section.

(Lines 477–478)

Comment:

4. Thank you for stating the following in the Competing Interests section:

Please confirm that this does not alter your adherence to all PLOS ONE policies on sharing data and materials, by including the following statement: "This does not alter our adherence to PLOS ONE policies on sharing data and materials.” (as detailed online in our guide for authors http://journals.plos.org/plosone/s/competing-interests). If there are restrictions on sharing of data and/or materials, please state these. Please note that we cannot proceed with consideration of your article until this information has been declared.

Please include your updated Competing Interests statement in your cover letter; we will change the online submission form on your behalf.

Response:

Thank you for your advice. We have updated the Competing Interests section and added "This does not alter our adherence to *PLOS ONE* policies on sharing data and materials."

(Line 472–473)

Comment:

5. We note that you have indicated that data from this study are available upon request. PLOS only allows data to be available upon request if there are legal or ethical restrictions on sharing data publicly. For more information on unacceptable data access restrictions, please see http://journals.plos.org/plosone/s/data-availability#loc-unacceptable-data-access-restrictions.

In your revised cover letter, please address the following prompts:

a) If there are ethical or legal restrictions on sharing a de-identified data set, please explain them in detail (e.g., data contain potentially sensitive information, data are owned by a third-party organization, etc.) and who has imposed them (e.g., an ethics committee). Please also provide contact information for a data access committee, ethics committee, or other institutional body to which data requests may be sent.

We will update your Data Availability statement on your behalf to reflect the information you provide.

Response:

Our updated Data Availability statement is as follows:

Since our research dataset contains potentially sensitive information, we have ethical restrictions. Please contact the institutional review board of the ethics committee when requesting data.

Contact information for our ethics committee: The institutional review board of the ethics committee of the Japanese Association of Neuro-Psychiatric Clinics; Shibuya-ku, Yoyogi 1–38–2, Tokyo Metropolis, Japan, Postal Code 151–0053, Phone +81–3-3320–1423.

(Lines 180–186,412–421)

Comment:

5. PLOS requires an ORCID iD for the corresponding author in Editorial Manager on papers submitted after December 6th, 2016. Please ensure that you have an ORCID iD and that it is validated in Editorial Manager. To do this, go to ‘Update my Information’ (in the upper left-hand corner of the main menu), and click on the Fetch/Validate link next to the ORCID field. This will take you to the ORCID site and allow you to create a new iD or authenticate a pre-existing iD in Editorial Manager. Please see the following video for instructions on linking an ORCID iD to your Editorial Manager account: <https://www.youtube.com/watch?v=_xcclfuvtxQ>

Response:

The ORCID of Dr. Norio Yasui-Furukori, the Corresponding author, is 0000–0002–4414–3770.

Additional Editor Comments:

Comment:

Please review your reference list to ensure that it is complete and correct. If you have cited papers that have been retracted, please include the rationale for doing so in the manuscript text, or remove these references and replace them with relevant current references. Any changes to the reference list should be mentioned in the rebuttal letter that accompanies your revised manuscript. If you need to cite a retracted article, indicate the article’s retracted status in the References list and also include a citation and full reference for the retraction notice.

Response:

Based on the reviewer’s comments, we added 5 references to the revised manuscript. As far as we can confirm, no retracted article was found. If you notice that the retracted article is included in the reference list, please let us know in detail.

(We added references 8–9, 21, 48 and 49.)

Reviewers' comments:

Reviewer #1:

Comment:

There are a few references missing, which I expect to see in the revised version of the introduction or the discussion:

1) “Clinical and psychopathological features associated with treatment-emergent mania in bipolar-II depressed outpatients exposed to antidepressants” J Affect Disord. 2018 Jul;234:131-138.

2) “Incidence, prevalence and clinical correlates of antidepressant-emergent mania in bipolar depression: a systematic review and meta-analysis” Bipolar Disord. 2018 May;20(3):195-227.

3) “The prevalence and predictors of bipolar and borderline personality disorders comorbidity: Systematic review and meta-analysis” J Affect Disord. 2016 May;195:105-18.

Response:

Thank you for your valuable suggestions. We cited these references and added sentences in the introduction and discussion.

(Lines 90–93, 280–281)

Comment:

Besides, the authors should also consider the followings:

4) Most of the include covariates dealt with monotherapy, which does not really reflect the real-world setting in BD: please further comment about this issue and consider referencing the suggested reference too: “Prevalence and clinical features associated with bipolar disorder polypharmacy: a systematic review” Neuropsychiatr Dis Treat. 2016 Mar 31;12:719-35.

Response:

We appreciate your insightful comments. As you noted, in real-world clinical practice, a polypharmacy approach is often used for patients with bipolar disorder.

Previously, our research group reported the use of polypharmacy in a series of collaborative studies, and we found that severe illness and an intractable disease course were significantly associated with the number of psychotropic drugs in real-world clinical settings (“Estimated model of psychotropic polypharmacy for bipolar disorder: Analysis using patients' and practitioners' parameters in the MUSUBI study” Hum Psychopharmacol. 2021 Mar;36(2):e2764. doi: 10.1002/hup.2764.).

We performed multivariate analysis with the presence or absence of various psychotropic drugs (mood stabilizers, antipsychotics, antidepressants, hypnotics) as independent variables in the present study. In addition to these factors, adding the presence or absence of polypharmacy of the above drugs as an independent variable causes potential statistically multicollinear problems because each factor becomes a nested structure. For this reason, the risk of manic/hypomanic episodes due to polypharmacy was not fully understood. Based on the comment, I added a statement to the limitation section.

(Lines 388–398)

Reviewer #2:

Comment:

- The article's title is "Real-world clinical predictors of manic episodes among outpatients with bipolar disorder". However, as described by the authors in their limitations, manic and hypomanic episodes are summed up together. This is a relevant information, and the presented results should be interpreted differently (i.e., 29.1% of the total sample experienced one manic OR one hypomanic episode during the follow-up year), since the two conditions could be quite different in terms of severity and prognosis. The authors should at least:

1) Present this clarification since the beginning; 2) Substitute the concept "manic episode" with "manic/hypomanic episode" throughout the text; 3) Provide, if possible, the exact number of people who experienced a manic episode; 4) Provide an explanation of their choice.

Response:

Thank you for your valuable suggestions. We changed "manic episodes" to "manic/hypomanic episodes" in the title and manuscript. We also explained that manic and hypomanic episodes were summed. We did not determine the exact number of people who experienced manic episodes over the course of one year from baseline (not hypomanic episodes). The manuscript has been revised to note the research limitations.

(Lines 383–386. And please ensure that the revised manuscript has been changed from "manic episodes" to "manic/hypomanic episodes".)

Comment

- In the section Matherials and Methods, the authors write that "a questionnaire was administered at 176 of the 1665 outpatient clinics belonging to the JAPC". Could you please further explain why only a portion of clinics was included in the study? If the original project aimed to include a larger portion, I think that this data could be better presented in the "Results".

Response:

Thank you for your advice. Doctors who worked at 176 psychiatric clinics, had particular interest in bipolar disorder, and belonged to JAPC were included in this study. The number "1665" indicates the scale of JAPC, and it is considered that the participation rate of the JAPC did not have a significant impact on this research. The description in the "Subjects and Methods" section has been revised to avoid confusion.

(Line 115)

Comment:

- Which questionnaire did the psychiatrists complete? Was it a structured/semi-structured one?

Response:

We created an original semi-structured questionnaire to collect the information provided in the results section and received responses from psychiatrists at the psychiatric clinics. The questions are described in the method section.

(Line 122)

Comment:

- Throughout the text the authors do not refer to any inclusion/exclusion criteria (except for BD NOS). Did the authors apply any other inclusion/exclusion criteria (i.e., age, medical comorbidities, ...)? Please, further explain this point in the section "Matherials and Methods".

Response:

Thank you for your valuable feedback. The inclusion/exclusion criteria were added to the Materials and Methods. Among the patients treated for bipolar disorder, we included patients with bipolar disorder who went to the outpatient department of the psychiatric clinic in the study. In addition to BD NOS, patients with serious physical conditions such as terminal cancer or intractable diseases, and cases with missing values in the questionnaire responses were excluded from the study. Patients were not excluded due to other factors.

(Lines 164–167)

Comment:

- In the section Results, I had some troubles in understanding a couple of ORs. In particular: 1) GAF Score, OR: 1.54 = people with higher scores had 54% higher odds of experiencing a manic/hypomanic episode vs people with lower scores? If so, since you splitted the GAF score variable in four groups, how much low is low? Moreover, if my interpretation is correct, please consider to rephrase the corresponding line in the Results; 2) BD type, OR: 0.55 = I think that is BD-II vs BD-I. Please see the point above.

Response:

Thank you for your valuable suggestions.

1) The odds ratio in the binomial logistic regression analysis was different from the risk ratio in the cohort studies. The odds ratio can only compare how influential the individual independent variables are. In our study, the dependent variable was a nominal variable, so we replaced it with a dummy variable; manic/hypomanic = 1, no manic/hypomanic = 0. Additionally, since the GAF score of the independent variable is based on an ordinal scale, we replaced it with a dummy variable, GAF (81–100) = 0, GAF (61–80) = 1, GAF (41–60) = 2, GAF (<41) = 3. The larger the GAF score dummy variable is, the lower the actual GAF score (the actual GAF scores and the dummy variable of GAF scores in this study are inversely correlated). Therefore, the GAF score OR = 1.54 (p<0.001) can be interpreted as a significantly lower GAF score in the manic/hypomanic group. The mean dummy variables of the GAF score were 43% higher in the no manic/hypomanic group than in the manic/hypomanic group. It was thought that the odds ratio was not adequately approximated to the risk ratio because the event rate exceeded 10%.

(Lines 156–157, 400–402)

2) Bipolar type was a nominal variable, and dummy variables were specified as BD-I = 1 and BD-II = 2 in this study. Therefore, in binomial logistic regression analysis, if there were more BD-I patients with manic/hypomanic episodes than BD-II patients, the odds ratio would be smaller. Bipolar type OR = 0.55 can be interpreted as having more BD-I patients with manic/hypomanic episodes than BD-II patients with manic/hypomanic episodes.

(Lines 161–162, 216–217)

Comment:

- Table 1 is not displayed correctly in my copy. Please fix this issue, if possible.

Response:

Thank you for your advice. We have confirmed the contents of Table 1. Request manuscript.docx from the Editor and try viewing it in Draft View from the "View" tab.

In Word, tables that run off the manuscript page can be seen using Draft View.

Comment:

- In the discussion (page 28, line 9), the authors refer to some effect sizes (manic state=4.54; rapid cycling=3.86; mixed features=2.32) as particularly large ones. However, according to the following manuscript (Henian Chen, Patricia Cohen & Sophie Chen (2010) How Big is a Big Odds Ratio? Interpreting the Magnitudes of Odds Ratios in Epidemiological Studies, Communications in Statistics - Simulation and Computation, 39:4, 860-864, DOI: 10.1080/03610911003650383), the considered ORs are smaller in size. Could you please double-check this, also according to the event rate in the exposed/unexposed groups?

Response:

Thank you for your important suggestion. According to the manuscript identified by the reviewer, the odds ratio of the manic state and rapid cycling found in our research is “medium”, not “large”. The sentence was corrected. Additionally, the odds ratio may not be adequately approximated to the risk ratio because the event rate of manic/hypomanic episodes is greater than 10%. Regarding this point, we cited the literature and added a sentence in the Limitations section.

(Lines 264, 400–402)

Comment:

- People with personality disorder are more likely to experience a manic/hypomanic episode. Since personality disorders is a noun under which is included a large variety of clinical conditions, is it possible to present a more stratified information (i.e., splitting into cluster A, B, or C), in order to provide a more focused and useful message to the reader?

Response:

Thank you for noting this important issue. Personality disorder is a diverse group of diseases, and identifying subclasses may be beneficial to the reader. However, this study did not subdivide patients, such as by classifying clusters of personality disorders. We added this point to the limitations section.

(Lines 399–400)
